# Committee Report

**Published:** 1891-02

**Authors:** D. E. Salmon

**Affiliations:** Chairman of Committee


### REPORT OF THE COMMITTEE ON ANIMAL DISEASES AND ANIMAL FOODS.

---

AMERICAN PUBLIC HEALTH ASSOCIATION.

*D. E. Salmon, Chairman.*

---

Your Committee in presenting its final report desires to call attention to the rapid progress of the last few years in elucidating the nature of the more serious diseases of animals and in preventing their ravages. Within seven years the bacilli which cause the two most common diseases of swine have been discovered and carefully studied. The protozoa which apparently cause Texas fever have also been demonstrated, as well as the fact that their development in the animal body is in some way connected with the ticks that are transported by cattle from the infected district. These discoveries clear up many of the mysterious characters which for a long time excited the astonishment and frequently the sceptism of scientific men in regard to the clinical observations that were from time to time made public.

Then we have seen the successful results of a still more advanced line of researches, *i. e.*, the study of the chemical products which accompany bacterial multiplication, their application for the production immunity, and even, in one case, the making by synthetical combinations in the laboratory of a compound which grants immunity. This at present appears to be the ideal achievement—the *ne plus ultra* of what we can hope for in the treatment of the animal body to produce immunity from disease.

It is a line of investigation which will sooner or later be applied to all of the non-recurrent diseases it is destined to be of the greatest service.

Your Committee is also gratified at the rapid progress being made in the United States in the eradication and control of animal diseases. The contagious pleuro-pneumonia of cattle, which four years ago threatened to extend over the whole country has been repressed and eradicated by the efforts of the general government, and so thorough has been the work that but two herds have been affected by it during the last three months. It is now only a question of a few months when the contagion will be entirely eradicated from the American continent. Texas fever has also been so thoroughly controlled that but few cases have occurred during 1890 in the country, the marked decrease being indicated by the summer insurance rates on export cattle which have on this account been reduced the present year about fifty per cent.

The infectious diseases of swine, tuberculosis of cattle and cholera of fowls are now the principal widespread and destructive plagues which carry on their ravages without control among the food producing animals of the United States. What action will be taken in regard to these by the national and State governments it is yet too early to predict, but it seems evident that the general demand for a more rigid and systematic inspection of meats will lead not only to the adoption of inspection, but will also eventually bring about general measures for the prevention of these diseases.

The subjects of animal diseases and animal foods are inseparably connected so far as the questions considered by your Committee are concerned. With the preventable diseases of meat-producing animals eradicated or controlled, and with a proper inspection at the time of slaughter, the animal food of this country would be beyond reproach or criticism. With a generally salubrious climate, with the finest pastures in the world, with that peculiarly American staple, maize, for feeding, in addition to the forage available in other countries, with a complete freedom from some of the worse plagues which affect the animals of the old world, with the most improved breeds which the world affords and with the most intelligent farmers found in any country, there is every reason why the meat of the United States should be superior to that produced elsewhere. Your Committee believe that the meat sold in this country has already attained to this condition of superiority, but they find that more thorough inspection and the repression of the animal plagues above mentioned are nevertheless desirable, and of great importance as sanitary measures. With the wealth and civilization of the United States it is felt that we should lead the world in taking such precautions rather than to follow in the wake of the most advanced government.

Referring to its former reports for greater details, your Committee closes its labors with this general survey of the important questions which you have placed in its charge for examination and report. The members individually and collectively return their thanks for the honor which has been conferred upon them, and for the trust and confidence reposed in them by this Association. As a Committee it has been our endeavor to point out the dangers with which our people are threatened through their food supply, and the means of removing them, but at the same time it has been necessary for us to use such moderate and conservative language as would guard against undue alarm among the masses of our people consuming so important a part of their daily nourishment. If we have accomplished something towards the dissemination of more intelligent ideas in regard to the diseases of animals, and more definite views as to the necessity of pure food and the means of obtaining it, we shall consider ourselves fortunate and well repaid for our labors.

Respectfully subbmitted,

D. E. Salmon,

*Chairman of Committee.*

